# Quo vadis, colesterol de las LDL?

**DOI:** 10.1515/almed-2023-0049

**Published:** 2023-05-30

**Authors:** Andrés Cobos, Pedro Valdivielso

**Affiliations:** UGC de Laboratorio, Hospital Universitario Virgen de la Victoria, Málaga, España; Departamento de Medicina y Dermatología, Servicio de Medicina Interna, Hospital Universitario Virgen de la Victoria, Universidad de Málaga e Instituto de Investigación Biomédica de Málaga (IBIMA-Plataforma BIONAND), Málaga, España

**Keywords:** colesterol de las LDL, Consenso Español, perfil lipídico básico

La prevención cardiovascular pasa por el control de los factores de riesgo, siendo uno de ellos, de naturaleza etiológica, los niveles de colesterol de LDL (cLDL). Hace pocos años, hubo que llamar la atención sobre la necesidad de conocer los niveles de cLDL y tomar decisiones terapéuticas en base a ellos y no a los niveles de colesterol total. Afortunadamente, este conocimiento ha calado, no sólo en las guías de práctica clínica, sino también entre los profesionales. No hace muchos años, también existían limitaciones para medir el colesterol de las HDL (cHDL), ya que en algunos centros sólo se medían cuando existían anomalías en los otros parámetros o sólo se medían en el ámbito hospitalario.

Afortunadamente, estas limitaciones han desaparecido y el avance del conocimiento permite ahora acceder a una amplia gama de parámetros lipídicos. La información liberada por los laboratorios clínicos debe responder no sólo a la petición del facultativo, sino que debe tener fundamento científico y ser clínicamente relevante. Con estos fines, el Grupo Multidisciplinar de Trabajo de Lípidos y Riesgo Vascular, formado por representantes de 13 Sociedades Españolas, presentan en este número un “Documento de Consenso para la determinación e informe del perfil lipídico en laboratorios clínicos españoles”, firmado por Arrobas et al. [1].

Este documento narra aspectos claves en la determinación de los lípidos a tener en cuenta, tanto en la fase preanalítica (ayunas o no, posición en el momento de la extracción, contexto clínico del paciente) y post analíticas. El documento describe con nitidez que elementos básicos deben estar incluidos en lo que denominan perfil básico y que se concreta en informar de las concentraciones séricas de colesterol total, triglicéridos, colesterol de HDL, colesterol de LDL (estimado) y colesterol-no-HDL. Con esta información elemental, es posible calcular el riesgo vascular de los pacientes y verificar el grado de control de la dislipemia y conocer si se han alcanzado los objetivos terapéuticos requeridos. Valores muy elevados o muy bajos de colesterol, triglicéridos o de colesterol de HDL abren la puerta al clínico para investigar la posible presencia de una dislipemia monogénica [2]. Así, otro de los aspectos relevantes del documento es resaltar, en texto y en anexos, que las hiperlipemias pueden ser además de un factor de riesgo una enfermedad en sí misma. Introducir el puntaje de las Clínicas de Lípidos Holandesas para el caso de la hipercolesterolemia familiar o el *score* de Moulin para la sospecha de síndrome de quilomicronemia familiar ante un caso de hipertrigliceridemia grave, es todo un acierto.

Con el mismo acierto, el documento se centra en comentar aspectos básicos en la estimación del cLDL, sabiendo claramente que el método de referencia, la cuantificación beta por ultracentrífuga es inaccesible salvo para laboratorios de investigación. El problema con la estimación del cLDL es que se deja influenciar por los niveles de triglicéridos, de ahí que el documento proponga, siguiendo la literatura, hasta tres fórmulas: la tradicional de Friedewald, la ecuación de Martin-Hopkins y la fórmula de Sampson. Estas tres estimaciones del cLDL han sido comparadas no sólo en relación con los niveles de triglicéridos sino también en su fiabilidad con los niveles muy bajos de cLDL, que hoy en día conseguimos con la triple terapia [3], aunque hay voces que indican que en la mayoría de los pacientes se pueden usar cualquiera de las tres fórmulas [4]. Esta situación no sólo nos llevar a vivir con la imperfección en un parámetro esencial en la prevención cardiovascular [5], si no que puede llevar a confusión a los clínicos. Las alternativas a la estimación del cLDL son dos, bien señaladas en el documento, el colesterol-no-HDL y la apolipoproteina B. A favor del primero, sin lugar a dudas, el coste, ya que este es nulo. Pero hay más, las últimas guías de ESC 2021 [6] establecen que para estimar el riesgo de evento mortal o no a 10 años se utilice el c-no-HDL, en lugar del colesterol total o el cociente colesterol total/cHDL de guías más antiguas. Por si esto fuera poco, estas guías consideran el c-no-HDL como objetivo terapéutico (30 mg/dL superior al cLDL recomendado). La cuestión ahora es: si es barato, no se afecta por los niveles de triglicéridos, es útil para estimar el riesgo pues captura todo el colesterol de las partículas aterogénicas y es además un objetivo terapéutico, ¿por qué no sacar el c-LDL estimado del centro de la prevención cardiovascular y posicionar ahí el c-no-HDL?

Más allá de lo básico, el documento menciona también la posibilidad de cuantificar el colesterol de las partículas remanentes, por cierto, con toda la incertidumbre de que este parámetro está influido por el colesterol de las LDL. Es cierto que la estimación de partículas remanentes o del colesterol de remanentes aportan información acerca del riesgo vascular del paciente [7], en especial en casos de dislipemia mixta o de hipertrigliceridemia, pero su cuantificación parece tener escaso valor clínico [8]. Por tanto, con buen criterio no han sido incluidas en el perfil básico.

Mención especial merece la determinación de la Lp(a), que este documento contempla como básico (al menos una vez) pero no rutinario hasta que no haya un fármaco específico frente a esta lipoproteína. Llama la atención, sin embargo, que la Lp(a) se incluya como alerta cuando los niveles de la misma excedan los 120 mg/dL. Esta cifra podría parecer muy elevada habida cuenta que niveles >50 mg/dL incrementan el riesgo vascular de forma notable en sujetos con hipercolesterolemia familiar [9], y que en el ensayo con Pelacarsen (Horizon) los puntos de corte para ser incluido son dos brazos, uno>70 mg/dL y otro>90 mg/dL. Sin embargo, estos niveles están en consonancia con las recomendaciones de la Sociedad Española de Arteriosclerosis, para remitir a una Unidad de Lípidos aquellos pacientes con niveles de Lp(a) superiores a 117 mg/dL [10], derivadas del estudio poblacional de Copenhagen [11].

El documento de consenso también incorpora la inclusión de niveles de colesterol de LDL deseables en relación con el riesgo vascular, en lugar de los tradicionales valores de “normalidad”. Igualmente, de gran interés es incluir alertas que llamen la atención del clínico respecto de ciertos valores considerados críticos y que motivarían la profundización de nuevos estudios en el paciente o su remisión a una unidad especializada. Es obvio que la inclusión de alertas aumentará las derivaciones a unidades de referencia y, en el contexto actual, cobra aún más importancia la mención que en el documento se hace a la e-consulta. La telemedicina es aquel contacto, acto médico, entre un profesional sanitario y su paciente usando medios telemáticos, evitando la presencialidad. Por el contrario, la teleconsulta se define como la consulta síncrona o asíncrona cuyo objetivo es permitir el intercambio de información entre profesionales sanitarios omitiendo la distancia funcional o geográfica [12]. En este sentido, el documento de consenso enumera hasta 11 componentes que de forma ideal deberían incluirse en dicha e-consulta relacionada con el riesgo vascular. Las teleconsultas entre profesionales se van a ir desarrollando de forma progresiva al menos en las áreas hospitalarias, con el consiguiente beneficio para los pacientes y para los propios profesionales sanitarios, disminuyendo la tradicional brecha entre niveles asistenciales. En nuestro país existe experiencia de e-consultas en pacientes con insuficiencia cardiaca y fibrilación auricular [13], lo que permite ordenar mejor la asistencia clínica presencial.

El documento de consenso se completa con abundante material suplementario, que compila información técnica sobre la variabilidad analítica, causas clínicas y farmacológicas de dislipemia y los antes mencionados cuestionarios para la hipercolesterolemia familiar y el síndrome de quilomicronemia familiar.

En su conjunto es un documento de valor que ayudará a los laboratorios clínicos y a los clínicos a emitir y recibir información muy útil para la prevención vascular y para el diagnóstico de las dislipemias.
